# Vitamin D and Tumor Necrosis Factor-Alpha Receptor Gene Polymorphisms in Various Hepatitis B Clinical Conditions in Turkey

**DOI:** 10.4021/gr544e

**Published:** 2013-10-31

**Authors:** Semra Tuncbilek, Kemalettin Aydin, Kenan Hizel

**Affiliations:** a Infectious Diseases and Clinical Microbiology Department, Ufuk University Medical School, Turkey; b Infectious Diseases and Clinical Microbiology Department, Karadeniz Technical University Medical School, Turkey; c Infectious Diseases and Clinical Microbiology Department, Gazi University Medical School, Turkey

**Keywords:** Hepatitis B, Single nucleotide polymorphism, TNF-α, Vitamin D, Ethnicity

## Abstract

**Background:**

The aim is to define the role of single nucleotide polymorphism on the progress of hepatitis B virus (HBV) infection. We evaluated polymorphisms of TNF-α-308, Vitamin D receptor Apa I and Taq I gene in patients with HBV infection.

**Methods:**

All subjects included were older than 18 years old. Sixty three patients had chronic HBV infection, 61 were HBsAg positive carriers and 59 were positive for anti-HBs and anti-HBc. Gene polymorphisms were evaluated by Amplification Refractory Mutation System PCR. For patients with chronic hepatitis, viral load, ALT levels, and histopathological evaluation of the liver were also compared.

**Results:**

Gender distribution was not different among groups; however, anti-HBs positive patients were significantly older than the other patients. ALT levels and viral load were significantly higher in chronic hepatitis group than the asymptomatic carriers group. Vitamin D receptor Apa I gene and Taq I gene and TNF-α -308 gene variant alleles were not different in all three groups. Variant alleles of three genes were not different in subgroups of chronic hepatitis patients formed according to ALT levels, viral load, histological activity index, and fibrosis score.

**Conclusions:**

Role of single nucleotide polymorphism in clinical status of various HBV infection states was not shown in this study. Considering the other studies performed with this aim, which strengthens the notion that ethnicity is an important factor, future studies with more patients from different ethnic groups may help to clear the role of polymorphisms in the clinical progress of HBV infection.

## Introduction

Developments in molecular genetics area induced the studies evaluating effect of the variability in human genome on immune response and disease mechanisms. Gene polymorphism is most commonly seen as single nucleotide polymorphism (SNP) in which one nucleotide is replaced with another. SNP can change the protein production coded by that gene or alter the biological function. Inheritance of these kinds of polymorphisms may cause the subject to be more susceptible or resistant to certain diseases [1]. Thus, clinical prognosis of an infectious disease can be determined beforehand by determining the genetic polymorphism. There are several gene products that define the antiviral immune reaction and host immune response [2].

Hepatitis B virus (HBV) can cause various clinical conditions from asymptomatic carrier to chronic necroinflammatory liver disease and hepatocellular carcinoma. The progress of the liver disease is defined with the immune response, which is shaped by interaction between host and virus. The mechanisms that define the viral clearance or become chronic are unknown. The host’s susceptibility to infectious diseases or severity of the infection cannot be attributed the virulence of the microbial agent solely. The very complex progress of the infection is determined by many factors that belong to both the virus and the host. For example, family studies conducted in China showed that HBe antigen positivity was more common in monozygotic than dizygotic twins, which suggested that the genetic factors of the host can determine the progress of the infectious disease [3].

The genes that possibly may have a role in HBV pathogenesis are tumor necrosis factor-alpha (TNF-α), interleukin (IL)-6, interferon-γ, IL-10, tumor growth factor-â, nitric oxide synthase, manganese superoxide dismutase and some other antioxidant enzymes, chemokine receptor 5, vitamin D receptor, estrogen receptor. A cytokine gene’s activity at transcriptional level is regulated by specific nucleotide variations on promoter area and variations on coding zone can change the function of the gene product.

TNF-α levels and TNF-α receptor regulation are increased in patients with HBV infection [4]. TNF-α gene polymorphism can determine the progress of HBV infection. TNF-α gene is located on MHC HLA class III region and two polymorphisms on promoter region -308 and -238 positions can change the expression of TNF-α. In German patients infected with HBV- 238 promoter variant is significantly correlated with HBV disease becoming chronic [5, 6].

Active form of Vitamin D is a hormone that has a role on calcium regulation and also has immune modulatory effects. It activates Th2 response while inhibiting Th1 response. Four genetic polymorphisms of vitamin D receptor are defined which are related with various immune diseases [7]. Moreover, one of the polymorphisms of vitamin D receptor gene is related with HBV clearance [8].

Although there are numerous studies evaluating the polymorphisms of these genes, the role of these polymorphisms in HBV infection progress and their relationship with each other has not been evaluated in detail yet. Moreover, considering the fact that these polymorphisms show variability between races, it is not possible to generalize the results of these studies.

In this study, we evaluated the TNF-α-308, VDR Apa I and VDR Taq I gene polymorphisms in asymptomatic HBsAg carriers, chronic HBV infection, compared with the results of anti-HBs positive people as control group.

## Materials and Methods

Subjects aged 18 years and over are informed about the study and samples, which were taken for routine purposes, of the consented subjects, were used in this study. Sixty three chronic hepatitis B patient, 61 inactive carriers (HBsAg were positive, liver transaminase levels were normal, and/or biopsy results were negative for necroinflammatory activity) and 59 anti-HBs and anti-HBc positive control subjects were included the study.

Demographic characteristics, medical history, previous laboratory test results (if present), concomitant diseases and medications of the eligible subjects were recorded. Liver enzymes of asymptomatic carriers and chronic hepatitis patients were evaluated. Viral load is determined by real time PCR.

Liver biopsy samples were evaluated and classified according to Modified Knodell Histological Activity Index [9, 10].

TNF-α, VDR Apa I and VDR Taq I gene polymorphisms were evaluated by “Amplification Refractory Mutation System-Polymerase Chain Reaction” (ARMS-PCR) method.

### Vitamin D receptor polymorphism

Genomic DNA was prepared from whole blood according to standard methods [11]. VDR genotypes denoted BB, Bb, and bb were determined after Bsm 1 restriction cleavage of genomic DNA amplified by PCR, as previously reported [11, 12]. A 740-bp fragment of the VDR gene, including the Apa I and Taq I restriction sites in intron 8 and exon 9, was amplified using specific primers 5’- CAGAGCATGGACAGGGAGCAA-3’ and 5’-GCAACTCCTCATGGCTGAGGTCTC-3’ [13]. Amplifications were performed in a reaction volume of 50 µL, by using 10 mM Tris-HCl (pH 9.0), 50 mM KCl, 1.5 mM MgCl_2_, 0.1% Triton X-100, 200 nM dNTP, 50 pmol/ µL of each primer and 1.25 units of Taq polymerase. All PCR reactions were carried out for 30 cycles at 94 °C for 1 min, at 68 °C for 1 min, at 72 °C for 1 min won a thermal cycler (MJR PTC100). The PCR products were digested with Apa I (10 U/µL at 37 °C) or Taq I (10 U/µL at 65 °C) and electrophoresed in a 2% ethidium bromide-agarose gel. Apa I digestion reveals genotypes denoted AA (496 bp), Aa (496, 279, and 217 bp), or aa (279 and 217 bp) and Taq I genotypes denoted TT (496 bp), Tt (496, 294, and 202 bp), or tt (294, and 202 bp) [13].

### Detection of the TNF-α polymorphism

An ASPCR was developed to detect the G to A transition polymorphism at position -308 of the TNF-α gene [14]. Four primers with a concentration of 30 pmol/µL, were used for the ASPCR: the 3’ primer (C1: position -144/-164: 5’- TCTCGGTTTCTTCTCCATCG-3’) was used in combination with either the 5’ primer (C2, position -328/-308G: 5’- ATAGGTTTTGAGGGGCATGG-3'), complementary to the TNF- α 1 allele, or the 5’primer (C3, position -328/308A: 5’- ATAGGTTTTGAGGGGCATGA-3’), which is complementary to the TNF-α 2 allele. Internal control primer: 5’-GAGTCTCCGGGTCAGAATGA 3’). After several laps of amplification, formation of a PCR product indicates the presence of the specific allele in the template DNA. Genomic DNA prepared as described earlier [15] was used for amplification in a final volume of 50 µL containing 50 mM KCL, 10 mM Tris-HCL , 2 mM MgCl_2_. Reactions were carried out for 30 cycles: 94 °C for 30 s, 58 °C for 60 s and 72 °C for 60 s on a thermal cycler (MJR PTC100). Denaturation was completed in first cycle at 94 °C for 5 min, and last cycle 72 °C for 5 min. Amplification products were analyzed by 2% agarose gel electrophoresis.

### Ethics

The study was approved by the Institutional Ethical Committee and informed consent was obtained from all recruited subjects. The procedures followed were in accordance with the ethical standards of the responsible committee on human experimentation and with the Helsinki Declaration of 1975, as revised in 2010.

### Statistical analysis

The frequencies of Apa I, Taq I and TNF G308A alleles were compared with chi square or fisher exact test.

Continuous variables such as age, viral load etc. were compared with Kruskal-Wallis test or Mann Whitney U test for comparing median values.

For all tests, a two-tailed P-value of 0.05 was considered as significant level. The analysis was performed by SPSS software (version 15.0, SPSS Inc. Chicago, USA).

## Results

The demographic characteristics of study patients are presented in Table 1. A total of 183 patients (59 anti-HBs positive, 61 asymptomatic carriers and 63 chronic hepatitis B patients) result were evaluated during this study. The distribution of the patients’ genders were not significantly different among groups (P = 0.725). However, the average age of the anti-HBs positive group was significantly higher than the other two groups (P < 0.001 for both groups).

**Table 1:** Subject Demographics and Some Laboratory Test Values

Variable	Anti-HBs (+)	Hepatitis B carrier	Chronic hepatitis
Gender (F/M)	27/32	24/37	25/38
Age (years; mean ± SD)	54.5 ± 15.3*	35.8 ± 12.3	36.6 ± 11.75
ALT (U/L; range, n)	-	8 - 74 (34)	14 - 394* (57)
Viral load (copy/mL; range, n)	-	-	70 - 6.27 × 108 (63)

* P < 0.001.

ALT levels and viral load of chronic hepatitis B infection group were significantly higher than the levels obtained for asymptomatic carriers (P < 0.001).

Vitamin D receptor variant alleles for Apa I gene were grouped in three as AA homozygote, Aa heterozygote, and aa homozygote. The alleles for Taq I gene were grouped as TT homozygote, Tt heterozygote, and tt homozygote. Variant alleles of TNF G308A also grouped as GG homozygote, GA heterozygote, and AA homozygote.

ALT level (≤ 40 or > 40 U/L), viral load (≤ 10,000 or > 10,000 copy/mL), HAI (≤ 5 or > 5), and fibrosis score (≤ 2 or > 2) were not different in patients with different alleles of Apa I, Taq I, or TNF G308A genes.

The variant alleles for all three genes showed no statistically significant difference regarding the clinical condition (chronic hepatitis, asymptomatic carrier or anti-HBs positive) (Table 2).

**Table 2:** TNF-α, VDR Apa I and Taq I Polymorphisms in the Investigated Groups

	Apa I Allele n(%)	
	AA homozygote	Aa heterozygote
Anti-HBs (+)	16 (27.1)	40 (67.8)
Hepatitis B carrier	24 (39.3)	28 (45.9)
Chronic hepatitis B	22 (34.9)	36 (57.1)

	Taq I Allele n(%)	
	TT homozygote	Tt heterozygote
Anti-HBs (+)	29 (49.2)	27 (45.8)
Hepatitis B carrier	22 (36.1)	31 (50.8)
Chronic hepatitis B	29 (46.0)	28 (44.4)

	TNF G308A Allele n(%)	
	GG homozygote	GA heterozygote
Anti-HBs (+)	52 (88.1)	7 (11.9)
Hepatitis B carrier	50 (82.0)	11 (18.0)
Chronic hepatitis B	48 (76.2)	14 (22.2)

## Discussion

In this study, there were no significant differences between various clinical states of HBV infection (asymptomatic carrier or chronic infection) and anti-HBs positive subjects regarding variability of alleles of TNF-α G308A gene and vitamin D Apa I and Taq I genes. Moreover, no statistically significant difference was obtained during this study when the variability of these alleles was compared in chronic hepatitis patients according to ALT level, viral load, and HAI. When evaluated together with other studies performed on volunteers from different races, even though negative, these comprehensive data strongly suggest that there are racial differences regarding the correlation of the immune response to HBV infection with these polymorphisms. The examples of these studies are discussed further below.

TNF-α, is an important cytokine defining the immune response of the host to HBV and viral clearance. In various studies, it has been reported that TNF-α gene polymorphism can determine the progress of HBV infection. The scientific literature on this subject has contrary results, the studies performed on Mongoloid suggesting that TNF-α G308A gene polymorphism may affect the immune response to HBV infection. In a meta-analysis study, which evaluated 21 studies on TNF-α G308A gene promoter, results of 4,230 chronic HBV infection patients and 2,905 control subjects from various races were evaluated. According to the analysis by ethnicity, a significantly lower risk was associated with -308 variant genotypes of GA and AA in Mongoloid patients while Caucasian subjects had no significant associations [16]. In another meta-analysis, of the 12 studies evaluating the various alleles of TNF-α and effect of these allele’s polymorphism on the immune response to HBV infection, 10 of the studies included the analysis TNF-α G308A gene [17]. Only one of the five studies showing that -308 GG genotypes had a significantly higher risk of HBV persistence compared with those with GA or AA genotype was performed in Italy [18], supposedly performed on Caucasian subjects and the rest was from China or Korea [17]. Moreover, another study performed in South Indian population concluded that TNF-α G308 was strongly associated with chronic hepatitis B [19].

In addition to these results, two meta-analyses evaluating the association of polymorphisms of TNF-α and hepatocellular carcinoma risk also revealed similar results. According to these, TNF-α -308G/A, TNF-α -238G/A and TNF-α -863C/A polymorphisms may be associated with HCC among Asians [20]. TNF-α -308GG gene polymorphism is associated with a modest decrease in the risk of HCC [21].

Our study was performed on a Caucasian population, and no effect of TNF-α G308A gene polymorphism was shown on the prognosis of HBV infections. However, another study performed on Turkish population showed that TNF-α -308 G/G polymorphism was significantly higher in HBV-infected subjects compared to healthy controls [22].

Another study performed in Italian subjects suggests that although not determinative on HBV clearance, TNF-α G308A gene polymorphism may have a role on the prognosis of patients with chronic HBV infection [18].

Besides these controversial results, which are obtained from a limited number of subjects, there are other studies performed on Caucasian subjects from different countries that have parallel findings to our study [5, 23, 24]; these studies also suggested that TNF-α G308A gene polymorphism does not have an important effect on HBV pathogenesis.

A similar study which also conducted in patients with chronic HBV infection, spontaneously recovered patients and health controls in Iranian population, revealed similar results to our study. Although the TNF-α G308A gene polymorphism is more common in this population than other Caucasian or Far East populations, it has no association with development of chronic HBV infection [24].

There are controversial results from Far East also; in a study performed on Japanese population TNF-α G308A gene polymorphism shown to have no effect on development of hepatocellular cancer [5]. Similarly another study performed in Thailand evaluated the association of TNF-α -238, -308, and -863 with HBV infection and showed that -863 A/A or A/C genotype was associated with increased TNF-α levels in response to HBV infection and induced hepatocyte damage [24]. Similarly, a South Korean study showed that TNF-α -238 and -308 alleles were not different in clearance and persistence groups [25].

A review by Tayebi S and Mohamadkhani A evaluation of 21 original papers showed that three different results could be obtained regarding TNF-α -308 gene polymorphism. The authors stated that it was not possible to obtain a reliable result due to problems like small sample size, selection of subjects from groups with different genetic characteristics and different stages of HBV infection [26].

In this study, we found no correlation between various clinical states of HBV infection (asymptomatic carrier or chronic infection), ALT level, viral load, HAI and variability of alleles of vitamin D receptor Apa I and Taq I genes. Moreover the literature evaluating VDR gene polymorphisms’ effect on the immune response to HBV infection are very limited and far from being conclusive. According to a study performed on African patients, tt homozygotes were significantly underrepresented among HBsAg positive subjects, and suggested that persons with genotype tt may be resistant to persistence of HBV infection [27].

Several studies conducted in China by the same group of researchers that evaluate the Taq I and Fok I polymorphisms also suggested that VDR gene polymorphism has an influence on genetic susceptibility to HBV infection [8, 28-30].

Another study performed in India, showed that VDR a/a allele is associated with the severity of HBV-related liver disease and higher viral load. However, in this study allelic distribution of VDR genes among chronic hepatitis B patients and healthy controls showed no significant difference [31].

In another study performed on 250 Taiwanese chronic HBV carriers evaluated vitamin D receptor gene polymorphisms. The frequency of VDR Taq T/t in carriers with hepatitis flare(s) was significantly higher than those without, which suggests adverse clinical outcomes in HBV carriers. Similarly, it was also higher in HBeAg positive carriers than those carriers whose HBeAg was negative; which also supports the relationship with negative outcome. In this study, no association with the risk of hepatocellular carcinoma and evaluated gene polymorphisms was found [32].

In a study performed in Iran evaluating 3,700 blood samples suggests polymorphisms in the T/T allele of VDR is possibly associated with occult HBV infection, thus suggests that VDR and its functional polymorphisms are related to sensitivity and resistance of the immune system to HBV in patients with occult HBV infection [33].

A recent study reported that the CYP27B1-1260 promoter polymorphism is possibly associated with the persistence, but not susceptibility to HBV infection in Chinese HBV patients, and that the VDR Taq I polymorphism is not related to chronic HBV infection [34]; which is parallel with our results.

The literatures on VDR gene polymorphisms, although limited, also suggest that ethnicity may have a variable effect [35, 36]. More studies performed on larger number of subjects and different ethnic groups may provide results that are more conclusive.

In addition to these controversial results, the role of single nucleotide polymorphism role cannot be ruled out in chronic hepatitis. There are many factors determining the pathogenesis of chronic hepatitis, which of some are even unknown. Thus, it is difficult to show the role of SNP in the presence of these factors as it is impossible to standardize race, genetic and environmental factors. 
